# The potential of heparin-induced extracorporeal LDL/fibrinogen precipitation (H.E.L.P.)-apheresis for patients with severe acute or chronic COVID-19

**DOI:** 10.3389/fcvm.2022.1007636

**Published:** 2022-10-11

**Authors:** Beate Roxane Jaeger, Hayley Emma Arron, Wiltrud M. Kalka-Moll, Dietrich Seidel

**Affiliations:** ^1^Lipidzentrum Nordrhein, Mülheim, Germany; ^2^Department of Physiological Sciences, Faculty of Science, Stellenbosch University, Stellenbosch, South Africa; ^3^Institut für infektiologische und mikrobiologische Beratung (Infactio^®^), Bedburg, Germany; ^4^Institut tür Klinische Chemie und Laboratoriumsmedizin, Ludwig-Maximilians-Universität München, Munich, Germany

**Keywords:** H.E.L.P. apheresis, PASC, COVID-19, long COVID, SARS-CoV-2, heparin, fibrinogen, rheology

## Abstract

Patients with long COVID and acute COVID should benefit from treatment with H.E.L.P. apheresis, which is in clinical use for 37 years. COVID-19 can cause a severe acute multi-organ illness and, subsequently, in many patients the chronic illness long-COVID/PASC. The alveolar tissue and adjacent capillaries show inflammatory and procoagulatory activation with cell necrosis, thrombi, and massive fibrinoid deposits, namely, unsolvable microthrombi, which results in an obstructed gas exchange. Heparin-induced extracorporeal LDL/fibrinogen precipitation (H.E.L.P.) apheresis solves these problems by helping the entire macro- and microcirculation extracorporeally. It uses unfractionated heparin, which binds the spike protein and thereby should remove the virus (debris). It dissolves the forming microthrombi without bleeding risk. It removes large amounts of fibrinogen (coagulation protein), which immediately improves the oxygen supply in the capillaries. In addition, it removes the precursors of both the procoagulatory and the fibrinolytic cascade, thus de-escalating the entire hemostaseological system. It increases myocardial, cerebral, and pulmonary blood flow rates, and coronary flow reserve, facilitating oxygen exchange in the capillaries, without bleeding risks. Another factor in COVID is the “cytokine storm” harming microcirculation in the lungs and other organs. Intervention by H.E.L.P. apheresis could prevent uncontrollable coagulation and inflammatory activity by removing cytokines such as interleukin (IL)-6, IL-8, and TNF-α, and reduces C-reactive protein, and eliminating endo- and ecto-toxins, without touching protective IgM/IgG antibodies, leukocyte, or platelet function. The therapy can be used safely in combination with antiviral drugs, antibiotics, anticoagulants, or antihypertensive drugs. Long-term clinical experience with H.E.L.P. apheresis shows it cannot inflict harm upon patients with COVID-19.

## Introduction

In COVID-19 pandemic, the key question is: which therapeutic approach should be favored in order to save seriously sick patients? What kind of approach is suitable to prevent looming acute lung failure involving microthrombi and inflammation of the endothelium (1–5) as a result of an excessive immune response of the body when the host's first lines of defense have already failed? We know that SARS-CoV-2 uses the angiotensin-converting enzyme 2 (ACE2) receptor and the transmembrane serine protease 2 (TMPRSS2) as gateways (6–8) to infect cells of the alveolar epithelium (1–4) and endothelial cells in the lungs, heart, kidneys, intestines, and liver (5). This is why patients with coronary artery disease (9–12), hypertension (3, 13), diabetes (3, 13), or obesity (3, 13) exhibit a higher mortality risk as their receptor density is up-regulated (14). Moreover, the binding of the SARS-CoV-2 spike protein inhibits and down-regulates ACE2 function which in turn promotes the inflammatory response (6–8). Diabetes for instance increases thrombogenicity and hyperactivates platelets, and so does hypertension by increasing shear stress in the vessels (15–17).

Histological studies confirmed the presence of the virus in both cell types: alveolar epithelium and endothelial cells (1–5). Alveolar tissue and adjacent capillaries reveal massive inflammatory and procoagulatory activation together with cell necrosis, thrombi, and massive fibrinoid deposits (1–5, 18, 19). It results in the clinical picture of an obstructed gas exchange. The enlargement of the diffusion barrier limits the benefits of artificial ventilation and extracorporeal membrane oxygenation (ECMO) (20–23). In addition, the latter promotes the formation of radicals as a side effect (20–23).

The application of H.E.L.P. apheresis could significantly contribute to the restoration of microcirculation in the lungs and other affected organs. The method, developed by Seidel and Wieland in 1984, primarily for patients with severe hyperlipidemia or familial homozygous hypercholesterolemia (24–30), has not only been proven beneficial as an ultima ratio treatment of arteriosclerosis and its atherothrombotic sequelae, it also has been successfully applied in coronary heart disease (24–27, 31–33) to prevent and treat graft vessel disease following heart transplantation (33–39), acute thrombotic graft occlusion following aortocoronary bypass surgery (40), preeclampsia (41, 42), strokes (43–46), unstable angina pectoris (47), and hyperlipoproteinemia (a) (32). It exhibits anti-inflammatory effects in chronic, and also acute inflammatory processes of the endothelium in the micro- and macrocirculation (26–36, 40, 48, 49) and has anticoagulant and anti-inflammatory properties (25, 50, 51).

## Methodology

During H.E.L.P. apheresis, blood cells are first separated from plasma in the extracorporeal circuit, then 400.000 units of unfractionated heparin are added to the plasma, and the pH is lowered to 5.12 using an acetate buffer. That is the isoelectric point for the optimal precipitation of the apolipoproteins from LDL cholesterol, lipoprotein (a) [Lp(a)], and VLDL, which are precipitated in the precipitation filter together with fibrinogen. The excess heparin is adsorbed, and bicarbonate dialysis balances the pH again. The blood cells of the patients are reinfused in parallel with a saline solution (24, 50). The duration of treatment-−2 h on average—can be shortened or extended to meet individual needs (50).

## Rationale for H.E.L.P. apheresis

Patients with acute and long COVID-19 most probably will benefit from H.E.L.P. apheresis due to the following reasons:

1. It has no allocation problem and allows direct access to the entire macro- and micro-circulation owing to its extracorporeal access.
2. It uses 400.000 units of unfractionated heparin in the extracorporeal circuit, which was shown of being capable to bind SARS-CoV-2 spike protein (19, 52), and thereby could directly remove the virus and viral debris during viraemia.
3. The large quantity of unfractionated heparin allows the desolvation of forming microthrombi without a bleeding risk due to the heparin adsorber (50).
4. Heparin-induced extracorporeal LDL/fibrinogen precipitation (H.E.L.P.) apheresis removes about 50–70% of fibrinogen, the most important coagulation protein, within 2–3 h, that in turn immediately improves oxygen supply in the capillaries (50, 51).
5. In addition, it partially removes the precursors of both the procoagulatory and the fibrinolytic cascade by 35–50%, thus de-escalating the entire haemorheologic system (50). However, antithrombin III is only eliminated by 25% (50) ensuring minimized bleeding risk complications.
6. From the very beginning, H.E.L.P. apheresis is rheologically effective (30, 31, 33, 53): it increases myocardial (30, 53), cerebral (54), pulmonary blood flow rates, and coronary flow reserve (53). These effects facilitate oxygen exchange in the capillaries sustainably (51).
7. It removes cytokines such as interleukin (IL)-6, IL-8, and TNF-α, and reduces C-reactive protein (CRP) concentrations by more than 50% (41, 48, 49). The heparin adsorber completely eliminates endo- and ecto-toxins (48), so that the excessive inflammatory response, the so-called “cytokine storm”, can calm down (18, 19, 48, 49).
8. Heparin-induced extracorporeal LDL/fibrinogen precipitation (H.E.L.P.) apheresis has already been successfully applied for septic multi-organ failure in pilot studies by Bengsch et al. (48, 49). In modified form, it showed a successful outcome in the enterohaemorrhagic *E.coli* (EHEC) epidemic in patients suffering from the hemolytic-uraemic syndrome (HUS) (55).
9. Heparin-induced extracorporeal LDL/fibrinogen precipitation (H.E.L.P.) apheresis is an established, commercially available system (B. Braun AG, Melsungen, Germany) that has been in clinical use for 37 years. It is easy to handle and was shown to reduce complication rates in acute and chronic cardiac patients very effectively by 82–97% (27, 29, 30, 32, 34, 36). The long-term clinical experience with H.E.L.P. apheresis suggests, with a probability close to certainty, that it cannot inflict harm upon patients with COVID-19.
10. It does not remove protective IgM or IgG antibodies and does not affect leukocyte or platelet function. In the past, the therapy has been shown to be well-tolerated and safe during treatment with antiviral drugs, antibiotics, anticoagulants, or antihypertensive drugs.

## Background

In patients who are suffering from severe COVID-19, the computed tomography (CT) scan of the lungs shows ground-glass-like interstitial thickening (5), (which presumably leads to acute respiratory distress syndrome (ARDS). As a result of an excessive immune response, it appears uncontrollable. The advanced disease stage develops after the initial antiviral defense lines of the innate immune system—such as protective effects of interferons and secretory IgA on alveolar epithelium—have failed to eliminate the virus. The presence of SARS-CoV-2 viraemia is the prerequisite for humoral antibody synthesis of IgM and IgG subtypes. They could lyse virus-infected cells in the presence of complement factors. As far as we know, the nature and extent of the cellular immune response to viral antigens are almost entirely dependent on T-lymphocytes (56). The cell-mediated antibody-dependent cytotoxicity is T-cell-dependent and, currently, is being the subject of intensive virological and cell biological research.

In principle, intervention in the inflammatory cascade takes place as early as possible before the onset of the “cytokine tsunami” in order to prevent uncontrollable coagulation and inflammatory activity (18, 19) harming microcirculation in the lungs and other organs. This may be the case in COVID, for example, as this cytokine storm likely results in the presence of microthrombi found in patients suffering from COVID-19 (57). These microthrombi have the ability to block microcapillaries and hence, inhibit oxygen exchange and supply at various organs, resulting in the various symptoms of long COVID such as muscle fatigue, breathlessness, sleep impairment, and anxiety or depression (58). The phenomenon of a “cytokine storm” was first described in 1973 in graft-vs.-host disease (GvHD) following organ transplantation, and later in ARDS, sepsis, Ebola, avian flu H5N1, smallpox, systemic inflammatory response syndrome (SIRS), and now in COVID-19 (59).

Cytokines are proteins that coordinate and modulate cellular immune responses: they guide and activate leukocytes–in particular, T-lymphocytes and monocytes–to the site of inflammation where cytokine secretion is regulated by positive feedback. During a “cytokine storm”, leukocytes are activated to such an extent that the immune response seems inexorable. High concentrations of IL-1*ß*, IL-6, and IL-8 are expressed (18, 19, 59–61). Furthermore, IL-1*ß* and IL-6, together with TNF-α —the latter being mainly expressed by macrophages-direct systemic inflammatory effects such as the increase in body temperature and blood flow, capillary permeability, and leakage. Due to the complexity of regulation and orchestral functions, IL-6 plays a key role in the transition of mechanisms of innate to acquired immunity (60, 62). The CRP triggers IL-6 (61) and IL-6 links procoagulatory activation, especially triggering fibrinogen production in the liver **[**51**]**. Whenever the body's defense is not able to clear the virus from all sites, the inflammation may persist in macrophages, in vascular beds, or in the brain stem and chronify, as recently reviewed by Proal and VanElzakker (63) with the consequence of a wide range of long-lasting clinical symptoms and impaired host immunity. In recent years, Pretorius and Laubscher (64) proved the persistence of insoluble clots containing excess alpha2-Antiplasmin bound plasminogen fibrinogen and amyloid proteins, which results in hindered fibrinolysis in long COVID patients.

## Discussion: Effects of HELP apheresis

The anti-inflammatory effects of H.E.L.P. apheresis had been intensively investigated by Bengsch et al. (35, 36) in the nineties. It has been applied by them in pilot studies to successfully treat sepsis and septic shock patients with multiple organ failures. In 2012, we were able to rescue a patient with EHEC-induced HUS from her comatose state within hours, and from kidney failure within 2 days (55).

In the case of COVID-19, H.E.L.P. apheresis could be of immediate benefit because this extracorporeal system can reduce the trigger and effector of the overwhelming immune response in a simultaneous manner. The SARS-CoV-2, circulating cytokines, CRP, on top fibrinogen are reduced drastically, the latter by 50% within 2 h. As a result, the rheology of the pulmonary microcirculation will immediately be relieved—without reduction of the erythrocyte concentration. Fibrinogen is the effector of plasmatic coagulation and decisive determinant in microcirculation, plasma viscosity, and erythrocyte aggregability (51). Owing to the use of unfractionated heparin, the antithrombotic effect is maximal.

Previous studies using positron emission tomography in heart transplant patients showed that the median coronary blood flow rate remains significantly increased by 17.5% for 24 h after a single 2-h apheresis procedure. It increases by 27% under simulated exposure to the administration of adenosine (33). In principle, the decreased fibrinogen concentration causes rheologically significant effects and facilitates oxygen exchange. Plasma viscosity is reduced by an average of 19%, and erythrocyte aggregability is significantly decreased by 60% (33). In addition, the vascular endothelial growth factor (VEGF) and nitric oxide (NO) release are favorably influenced (33). The improvements have also been demonstrated for cerebral blood flow in the cardiac patients, where they profit from a 63% increase in the CO_2_ reserve capacity (54).

Heparin-induced extracorporeal LDL/fibrinogen precipitation (H.E.L.P.) apheresis reduces LDL cholesterol and Lp(a) concentrations with similar efficacy as fibrinogen (24, 25), thereby improving endothelial function (33, 53, 54). With regards to LDL reduction through apheresis, it remains unclear whether SARS-CoV-2 resembles delta coronavirus, which uses cholesterol as a vector due to its lipid envelope (65).

For practical reasons it is important to mention that H.E.L.P. apheresis is not restricted to a 2-h treatment time. The system can be recirculated for many hours—until the precipitate filter is saturated. The precipitate filter however can also be exchanged during the procedure, so the fibrinogen concentration theoretically could be reduced by up to 99.9999%. In-depth preliminary studies into the influence of H.E.L.P. apheresis on the kinetics of the procoagulation and fibrinolytic cascades have shown that the precursors of both cascades are also reduced by 35–50% within 2 h—with the exception of antithrombin III, which is reduced by 25% (50). Taking together, H.E.L.P. apheresis thus de-escalates the coagulation situation of both arms without any bleeding risk due to the complete adsorption of unfractionated heparin (50).

The heparin adsorber also has the ability to eliminate endo- and exo-toxins regardless of viral or bacterial origin (48, 49, 55). Recent data from Carlo Brogna indicate that the SARS-CoV-2 virus acts as a bacteriophage on the microbiome of the lungs and the guts of infected patients, thereby inducing the bacteria to produce neurotoxic “conotoxins”. These so-called conotoxins might also be eliminated by means of H.E.L.P. apheresis (64).

The use of H.E.L.P. apheresis should be considered for the treatment of patients with acute and long COVID in order to restore the vascular homeostasis, remove inflammatory and thrombogenic mediators, and to avoid unnecessary suffering. Our first experiences with patients with long COVID are promising and summarized in the corresponding article. Meanwhile, we could successfully treat hundreds of patients with long COVID with this method.

## Data availability statement

The original contributions presented in the study are included in the article/supplementary material, further inquiries can be directed to the corresponding author.

## Author contributions

BJ created the working hypothesis and wrote the paper. HA helped in editing, proofreading, and discussing the theory. WK-M helped brainstorm, discuss the theory, and refine it. DS was the inventor of the HELP apheresis helped with the theoretical hypothesis and editing. All authors contributed to the article and approved the submitted version.

## Conflict of interest

Authors BJ and DS filed a patent of the use of HELP Apheresis for long COVID to avoid misuse. The remaining authors declare that the research was conducted in the absence of any commercial or financial relationships that could be construed as a potential conflict of interest.

## Publisher's note

All claims expressed in this article are solely those of the authors and do not necessarily represent those of their affiliated organizations, or those of the publisher, the editors and the reviewers. Any product that may be evaluated in this article, or claim that may be made by its manufacturer, is not guaranteed or endorsed by the publisher.
